# Interactions between vitamin B_2_, the *MTRR* rs1801394 and *MTR* rs1805087 genetic polymorphisms, and colorectal cancer risk in a Korean population

**DOI:** 10.4178/epih.e2024037

**Published:** 2024-03-11

**Authors:** Madhawa Gunathilake, Minji Kim, Jeonghee Lee, Jae Hwan Oh, Hee Jin Chang, Dae Kyung Sohn, Aesun Shin, Jeongseon Kim

**Affiliations:** 1 Department of Cancer Biomedical Science, National Cancer Center Graduate School of Cancer Science and Policy, Goyang, Korea; 2 Center for Colorectal Cancer, National Cancer Center Hospital, National Cancer Center, Goyang, Korea; 3 Department of Preventive Medicine, Seoul National University College of Medicine, Seoul, Korea

**Keywords:** Vitamin B_2_, Methionine synthase, Methionine synthase reductase, Genetic polymorphism, Colorectal cancer

## Abstract

**OBJECTIVES:**

We explored whether the association between vitamin B_2_ and colorectal cancer (CRC) risk could be modified by the *MTRR* rs1801394 and *MTR* rs1805087 genetic polymorphisms and examined whether the interaction effects are sex-specific.

**METHODS:**

We performed a case-control study involving 1,420 CRC patients and 2,840 controls from the Korea National Cancer Center. Dietary vitamin B_2_ intake was assessed using a semiquantitative food frequency questionnaire, and the association with CRC was evaluated. Genotyping was performed using an Illumina MEGA-Expanded Array. For gene-nutrient interaction analysis, pre-matched (1,081 patients and 2,025 controls) and matched (1,081 patients and 1,081 controls) subsets were included. Unconditional and conditional logistic regression models were used to calculate odds ratios (ORs) and 95% confidence intervals (CIs).

**RESULTS:**

A higher intake of vitamin B_2_ was associated with a significantly lower CRC risk (OR, 0.65; 95% CI, 0.51 to 0.82; p<0.001). Carriers of at least 1 minor allele of *MTRR* rs1801394 showed a significantly higher CRC risk (OR, 1.43; 95% CI, 1.12 to 1.83). Males homozygous for the major allele (A) of *MTRR* rs1801394 and who had a higher intake of vitamin B_2_ had a significantly lower CRC risk (OR, 0.31; 95% CI, 0.18 to 0.54; p-interaction=0.02). In *MTR* rs1805087, males homozygous for the major allele (A) and who had a higher vitamin B_2_ intake had a significantly lower CRC risk (OR, 0.38; 95% CI, 0.25 to 0.60; p-interaction<0.001).

**CONCLUSIONS:**

The *MTRR* rs1801394 and *MTR* rs1805087 genetic polymorphisms may modify the association between vitamin B_2_ and CRC risk, particularly in males. However, further studies are warranted to confirm these interaction results.

## GRAPHICAL ABSTRACT

[Fig f2-epih-46-e2024037]

## Key Message

We conducted a case-control study to observe the association between vitamin B_2_ intake and the risk of colorectal cancer (CRC), and to determine whether this association could be modified by the methionine synthase (*MTRR*) rs1801394 and methionine synthase reductase (*MTR*) rs1805087 genetic polymorphisms. Higher intake of vitamin B2 is a protective factor in lowering CRC risk, and rs1801394 of *MTRR* and rs1805087 of *MTR* may particularly modify this association in males.

## INTRODUCTION

According to the GLOBOCAN 2020 estimates, colorectal cancer (CRC) is one of the most common cancers worldwide, accounting for 10% of all cancers [1]. Moreover, CRC is the second leading cause of cancer-related death and accounts for approximately 9.4% of deaths from all cancer types. In Korea, the latest data from 2019 show that CRC incidence and mortality rates are the fourth and third highest, respectively, among all cancers [2]. Thus, it is important to investigate the etiology and pathogenesis of CRC comprehensively [1]. Several etiological factors related to CRC occurrence, including diet and lifestyle, have been explored [3]. Based on the World Cancer Research Fund/American Institute for Cancer Research (WCRF/AICR) evidence, red and processed meats are associated with an increased risk of CRC, whereas whole grains, dietary fiber, dairy products and calcium supplements have a protective role against CRC risk [4]. In addition, specific micronutrients involved in one-carbon metabolism that are essential for maintaining DNA integrity have been shown to have a protective effect against CRC risk [5].

Vitamin B_2_ is the cofactor for 5, 10-methylenetetrahydrofolate reductase (MTHFR), which catalyzes the reduction of 5, 10-methyltetrahydrofolate (THF) to 5-methyl THF for subsequent DNA methylation reactions [6]. Inadequate levels of vitamin B_2_ could lead to derangements in one-carbon metabolism, resulting in high blood homocysteine levels [7,8]. Previous *in vitro* studies have suggested that higher plasma concentrations of homocysteine are associated with the rapid proliferation of tumor cells [9,10]. Several studies investigating the relationship between hyperhomocysteinemia and the risk of CRC have indicated that elevated homocysteine levels are associated with an increased risk of CRC [11-13]. Inverse associations between vitamin B_2_ intake and CRC risk have been observed in some studies [14,15]. However, these findings are not consistent across all studies, as no correlation between vitamin B_2_ intake and CRC risk was found in other research [16,17]. These contradictory results imply the involvement of additional factors, particularly genetic factors, in interactions affecting these relationships.

Single nucleotide polymorphisms (SNPs) in genes that encode enzymes related to folate metabolism contribute to the complexity of the association between vitamin B_2_ and CRC risk. Polymorphisms in the methionine synthase (*MTRR*) and methionine synthase reductase (*MTR*) genes might play major roles in CRC risk. A recent study reported that the GG genotype of *MTRR* rs1801394 can be a protective marker for CRC risk in Taiwan [18]. Other studies noted that the *MTRR* rs1801394 polymorphism might have a detrimental influence on the risk of CRC in patients with GG genotype compared to patients with AA genotypes [19,20]. The *MTRR* rs1801394 polymorphism is responsible for the substitution of isoleucine with methionine at codon 22 in the *MTRR* enzyme, yielding a variant protein exhibiting 4-fold lower activity than the wild-type protein *in vivo*. Thus, the *MTRR* rs1801394 G allele should decrease the availability of S-adenosylmethyonine (SAM) by reducing the level of active *MTR* to induce DNA hypomethylation, thereby modulating CRC risk [21]. The *MTR* rs1805087 GG genotype has been associated with an increased risk of CRC [22]. The *MTR* rs1805087 polymorphism can replace aspartic acid with glycine in the protein-binding region of methionine synthase, and this substitution leads to a less effective enzyme that promotes a modest reduction in homocysteine levels, which may have a protective effect [21]. However, there is a paucity of evidence related to the interactive effect of vitamin B_2_ and these 2 SNPs on CRC risk.

Thus, we aimed to observe the associations of vitamin B_2_ and the *MTRR* rs1801394 and *MTR* rs1805087 genetic polymorphisms with CRC risk. Moreover, we explored the interaction effects of vitamin B_2_ intake and the *MTRR* rs1801394 and *MTR* rs1805087 genetic polymorphisms on the risk of CRC and determined whether the interaction effects are sex-specific in a Korean population.

## MATERIALS AND METHODS

### Study population

**Figure 1.**
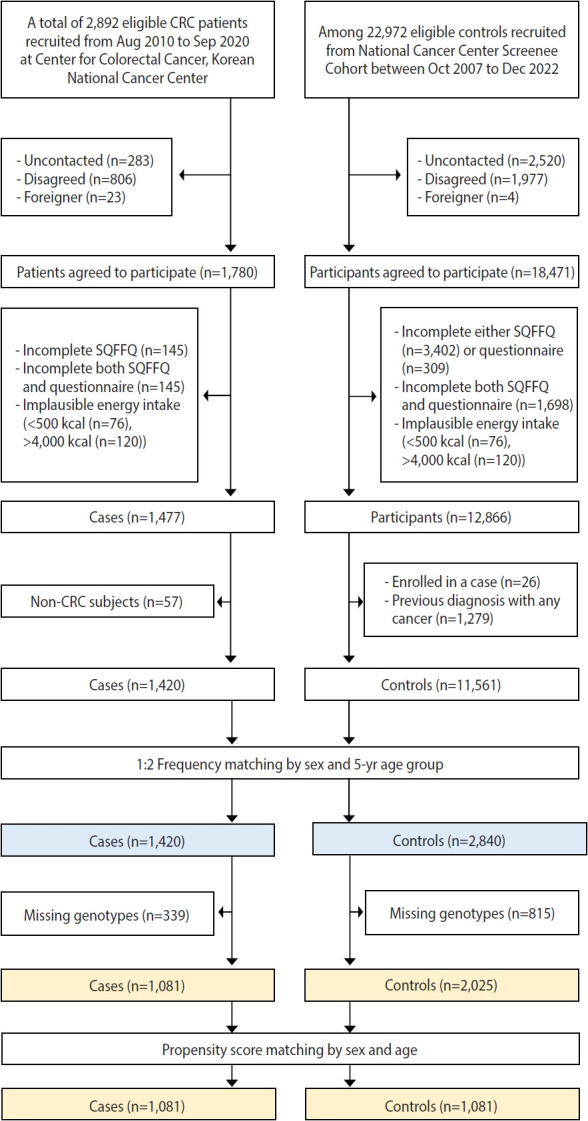
Selection of study population. CRC, colorectal cancer; SQFFQ, semiquantitative food frequency questionnaire.

The study participants were recruited from 2 research centers of the National Cancer Center (NCC) of the Korea. Patients were defined as those who newly diagnosed with CRC between August 2010 and September 2020 at the Center for Colorectal Cancer of the NCC. Of the 1,780 patients who agreed to participate in this study, 290 participants were excluded due to incomplete data from the semiquantitative food frequency questionnaire (SQFFQ) or general questionnaire, and 13 others were excluded due to implausible energy intake (< 500 or > 4,000 kcal/day). We also excluded 57 non-CRC patients. Thus, there were 1,420 eligible CRC patients for the study. The controls were selected from people visiting the Center for Cancer Prevention and Detection at the same hospital for the health check-up program provided by the National Health Insurance Cooperation from October 2007 to December 2022. Of the 18,471 controls, 5,409 participants with incomplete SQFFQ or general questionnaire data and 196 others with implausible energy intake (< 500 or > 4,000 kcal/day) were excluded. Participants were also excluded if they were enrolled in a case (n= 26) or previously diagnosed with any cancer (n= 1,279). Among the eligible participants, controls were selected by frequency matching to CRC patients by sex and 5-year age group (case:control ratio of 1:2). Finally, 1,420 cases and 2,840 controls were included in this study. Due to missing genotype data, including those without chip data and those with missing genotypes for the 2 target SNPs, 399 CRC patients and 815 controls were further excluded. The gene-environment interaction analysis was performed in both pre-matched and matched populations. For pre-matching analysis, 1,081 CRC patients and 2,025 controls were selected. We performed propensity score matching considering sex and age based on the nearest neighbor method with a 1:1 ratio using the “MatchIt” package in R software version 4.2.0 [23]. For matched analysis, 1,081 CRC cases and 1,081 controls were selected (Figure 1).

### Outcome assessment

The anatomical location of the CRC was determined according to the International Statistical Classification of Disease and Related Health Problems, 10th revision. The anatomical sites were subsequently categorized into 3 subgroups: (1) the proximal colon (including the cecum, ascending colon, hepatic flexure, transverse colon, and splenic flexure); (2) the distal colon (including the descending colon, sigmoid-descending colon junction, and sigmoid colon); and (3) the rectum (including the rectosigmoid colon and rectum).

### Data collection

Information on socio-demographic and lifestyle characteristics, including age, sex, weight, height, first-degree family history of CRC, supplement use, marital status, education level, monthly income, occupation, smoking status, alcohol consumption, and physical activity, was collected by well-trained interviewers using a structured questionnaire. Body mass index (BMI) was calculated as body weight (kg) divided by the square of height (m^2^). Dietary data were collected using a 106-item SQFFQ that was developed for Korean adults. The validity and reproducibility of the SQFFQ have been previously reported [24]. Participants were asked to provide their average food frequency (on a 9-point scale of never or rarely, 1 time/mo, 2-3 times/mo, 1-2 times/wk, 3-4 times/wk, 5-6 times/wk, 1 time/day, 2-3 times/day) and the average portion size (on a 3-point scale of small, medium, or large) for each food item during the previous year. The daily intake of vitamin B_2_ and total calories were calculated using a computer-aided nutritional analysis program (CAN-Pro 4.0, Korean Nutrition Society, Seoul, Korea).

### Genotyping

Blood samples were collected from each participant, and DNA was extracted using a MagAttract DNA Blood M48 Kit (Qiagen, Hilden, Germany) and BioRobot M48 automatic extraction equipment (Qiagen). Genotyping of the SNPs was performed using the Illumina MEGA-Expanded Array (Illumina Inc., San Diego, CA, USA) comprising 123,000 SNPs. Genotype imputation was conducted using the Michigan imputation server with the 1000 Genome Project phase 3 East Asian ancestry integrated variant set release GRch37/hg19 (https://www.1000genomes.org/) as a reference panel. We used SHAPEIT (v2.r837) for phasing and IMPUTE2 (2.3.2) for SNP imputation. After filtering for an INFO score over 0.6, the following quality control criteria were used for further exclusions: missingness for genotypes and individuals (genotype call rates) < 98%, SNPs with a minor allele frequency < 5%, and SNPs with Hardy‒Weinberg equilibrium p-value < 1 × 10^-6^. Consequently, both *MTRR* rs1801394 and *MTR* rs1805087 met the quality control criteria and were eligible for inclusion in the final analysis because of their minor allele frequencies of 0.28 and 0.14, respectively.

### Statistical analysis

The descriptive statistics are presented as the mean± standard deviation (SD) for continuous variables and as numbers (percentages) for categorical variables. A generalized linear model and the chi-square test were used to compare the differences in means and distributions of general characteristics of the study participants, respectively. The amount of dietary intake was divided into quartiles based on its distribution among the controls. The associations between vitamin B_2_ intake and CRC risk were assessed using unconditional logistic regression models to calculate odds ratios (ORs) and 95% confidence intervals (CIs). The lowest intake group (Q1) was used as the reference. The median value for each quartile category of vitamin B_2_ was used as a continuous variable to test for trends in the regression model. The multivariable logistic regression model considered potential covariates such as age (continuous), sex (male/female), BMI (continuous), first-degree family history of CRC (yes/no), supplement use (yes/no), marital status (married, single, divorced/widowed/other), education level (elementary school or less, middle school, high school, or college or more), monthly income (< 2, 2-4, or ≥ 4 million Korean won/mo), occupation (housewife, profession/office worker, sales/service, or agriculture/laborer/unemployed/other), smoking status (nonsmoker, former smoker, or current smoker), alcohol consumption (non-drinker, former drinker, or current drinker), physical activity (yes/no), red meat intake (continuous), and total energy intake (continuous). A stratified analysis based on anatomical subsites (proximal colon, distal colon, and rectal cancers) was performed using multinomial logistic regression models.

To observe the associations between *MTRR* rs1801394 and *MTR* rs1805087 genetic polymorphisms and CRC risk, we used 3 genetic models—namely, codominant, dominant, and recessive—with unconditional and conditional logistic regression models for pre-matched and matched populations, respectively. The 3 genetic models were tested in 2 statistical models: model I was a crude model, and model II was adjusted for potential confounding variables, including age, sex, BMI, alcohol consumption, smoking status, marital status, occupation, education, family history of CRC, supplement use, monthly income, regular exercise, red meat intake, and total energy intake. We also assigned a score for each individual depending on the number of minor alleles present in their genotypes for each SNP. Consequently, the 3 genotypes of both SNPs were weighted as follows: A/A= 0, A/G= 1, and G/G= 2, where a higher score represented a greater likelihood of increased CRC risk. Then, we summed the scores for both SNPs. The total score was standardized, and an association with the risk of CRC was observed. The interactions between vitamin B_2_ and selected candidate SNPs in relation to CRC were tested using logistic regression models via the likelihood ratio test in the dominant model. The regression models that were used for model I and model II were similar to the aforementioned models. For multiple testing correction, the false discovery rate adjustment was performed using the Benjamini-Hochberg procedure. All analyses were performed using SAS version 9.4 (SAS Institute Inc., Cary, NC, USA). A p-value < 0.05 was considered to indicate statistical significance.

### Ethics statement

This study was conducted according to the guidelines laid down in the Declaration of Helsinki and all procedures involving human subjects/patients were approved by the Institutional Review Board of Korea National Cancer Center (IRB No. NCC2021-0181). Written informed consent was obtained from all study subjects before participation.

## RESULTS

### Patient characteristics

Table 1 describes the general characteristics of the study population. The average age was 57.6± 9.5 years in the control group and 58.1± 10.2 years in the case group. Overall, the CRC patients had higher rates of first-degree family history of CRC, history of alcohol consumption, and higher marital status. Moreover, the individuals in the case group had lower supplement use, lower education levels, lower monthly income, lower professional occupation status, and lower participation in regular physical activity than the controls (p< 0.05). Stratification by sex showed a significantly greater mean BMI among female patients, while the male patients had a significantly lower mean BMI and smoking rate than the controls (p< 0.05). The distributions of other characteristics exhibited the same trend for both subgroups (p< 0.05). The results followed similar trends in pre-matched and matched populations used for gene-nutrient interaction analysis (Supplementaray Materials 1 and 2).

### Comparison of vitamin B_2_ intake

The dietary intake of vitamin B_2_ is presented in Table 2. In the overall population, the controls had higher intakes of vitamin B_2_ than the patients (p< 0.001). The mean total energy intake was greater in patients than in controls (p< 0.001). Stratification by sex showed the same trend in male and female subgroups (p< 0.001).

### Associations between vitamin B_2_ intake and colorectal cancer risk

The associations of vitamin B_2_ intake with CRC risk are shown in Table 3. A lower risk of CRC was observed in those who had a higher intake of vitamin B_2_ (OR _Q4 vs. Q1_, 0.65; 95% CI, 0.51 to 0.82; p for trend< 0.001) after adjustment for potential covariates. Moreover, higher vitamin B_2_ intake was significantly associated with a reduced risk of rectal cancer (OR _Q4 vs. Q1_, 0.40; 95% CI, 0.27 to 0.58; p for trend< 0.001).

In sex subgroups, the risk of CRC also tended to decrease with a high intake of vitamin B_2_ after adjustment for confounding factors for both males (OR _Q4 vs. Q1_, 0.60; 95% CI, 0.45 to 0.82; p for trend < 0.001) and females (OR _Q4 vs. Q1_, 0.60; 95% CI, 0.40 to 0.90; p for trend= 0.008). The effect of vitamin B_2_ on CRC incidence according to anatomical site exhibited a similar trend, with the strongest association occurring in patients with rectal cancer (males: OR _Q4 vs. Q1_, 0.40; 95% CI, 0.23 to 0.58; p for trend < 0.001; females: OR _Q4 vs. Q1_, 0.27; 95% CI, 0.12 to 0.60; p for trend < 0.001).

### Association between genetic polymorphisms and the risk of colorectal cancer

Supplementary Material 3 shows the associations between *MTRR* rs1801394 and *MTR* rs1805087 and CRC risk stratified by sex and anatomical site according to the codominant, dominant, and recessive models in the pre-matched population. None of the results were significant, although we found that there was marginal significance for those who carried at least 1 minor allele of *MTRR* rs1801394 for the risk of CRC with respect to the dominant model (OR, 1.16; 95% CI, 0.97 to 1.38). Even though the result was null, a marginally significantly greater risk was observed between a 1-SD increase in the risk score associated with the combination of 2 SNPs and CRC (OR, 1.05; 95% CI, 0.96 to 1.15). In the matched population, those who carried at least 1 minor allele of *MTRR* rs1801394 showed a significantly increased risk of CRC in the dominant model (OR, 1.43; 95% CI, 1.12 to 1.83). Based on the risk score, the association result was consistent with the pre-matched population (OR, 1.04; 95% CI, 0.94 to 1.15) (Supplementary Material 4).

### Interaction between genetic polymorphisms and the risk of colorectal cancer

In the matched population, males who were homozygous for the major allele (A) of *MTRR* rs1801394 and had a higher intake of vitamin B_2_ showed a significantly lower risk of CRC, with a significant interactive effect (OR, 0.31; 95% CI, 0.18 to 0.54; p for interaction= 0.02). Similarly, in *MTR* rs1805087, males who were homozygous for the major allele and had the highest vitamin B_2_ intake showed a significantly lower risk of CRC (OR, 0.38; 95% CI, 0.25 to 0.60) with a significant interaction (p for interaction < 0.001) (Table 4).

Comparatively, in the pre-matched population, males who were homozygous for major allele (A) of *MTRR* rs1801394 and had a higher intake of vitamin B_2_ had a significantly lower risk of CRC (OR, 0.48; 95% CI, 0.30 to 0.78; p for interaction= 0.02). For *MTR* rs1805087, males who were homozygous for major allele (A) with the highest vitamin B_2_ intake had a significantly lower risk of CRC (OR, 0.57; 95% CI, 0.38 to 0.84), with a significant interaction (p for interaction= 0.02) (Supplementary Material 5).

## DISCUSSION

The present case-control study investigated the interactive effects of dietary vitamin B_2_ intake and 2 genetic variants (*MTRR* rs1801394 and *MTR* rs1805087) on CRC development. A high intake of dietary vitamin B_2_ was associated with a decreased risk of CRC. There are significant synergistic effects between major allele carriers of *MTRR* rs1801394 and *MTR* rs1805087 genetic polymorphisms and high vitamin B_2_ consumption on CRC risk reduction, particularly in males.

Prior studies have reported associations between vitamin B_2_ intake and the risk of CRC, but the results are inconsistent. One prospective cohort analysis from the Women’s Health Initiative Observational Study indicated that total intake of vitamin B_2_ was associated with a reduced risk of CRC overall and regional spread of the disease [14]. A higher plasma concentration of vitamin B_2_ was associated with a lower CRC risk in the European Prospective Investigation into Cancer and Nutrition cohort [25]. Both of these findings are consistent with our results. However, a pooled analysis of the Nurses’ Health Study and the Health Professional Follow-up Study cohorts revealed that vitamin B_2_ intake was not associated with the risk of CRC, where the pooled relative risk and 95% CI for total vitamin B_2_ and dietary vitamin B_2_ intake in the highest quintile were 0.93 (95% CI, 0.81 to 1.06) and 0.89 (95% CI, 0.61 to 1.28), respectively [26]. In our study, we observed that the effect estimate for the highest quartile intake of vitamin B_2_ was 0.65. Consistent with our results, a large population-based case-control study conducted in Canada showed that the highest, compared to lowest, vitamin B_2_ quartile intake was associated with a decreased risk of CRC [15]. A case-control study in China reported an inverse association between dietary vitamin B_2_ intake and CRC risk [27]. Moreover, a recent case-control study showed that a decreased risk of CRC was associated with a greater intake of vitamin B_2_ [28]. In contrast, a case‒control study reported that there was no association between vitamin B_2_ and CRC risk [29].

Vitamin B_2_ may influence CRC risk through the one-carbon metabolism pathway because it is an essential coenzyme for MTHFR, the enzyme involved in homocysteine remethylation and DNA methylation [30,31]. Poor vitamin B_2_ status is also a known risk factor for certain cancers [32]. Several studies in experimental animals have shown that vitamin B_2_ deficiency may affect carcinogenesis. The formation of single-strand breaks induced by hepatic carcinogens was more pronounced in rats fed a vitamin B_2_-deficient diet [33]. Moreover, the induction of repair enzymes such as DNA ligase and DNA polymerase β was enhanced in vitamin B_2_-deficient rats. Since DNA damage and its subsequent repair may contribute to carcinogenesis, the modulation of these processes by vitamin B_2_ could affect metabolism in cancer. Oxidative stress, which is caused by an imbalance between free radicals and the antioxidant defense system, has been recognized as a contributing factor in the development of chronic diseases such as cancer, cardiovascular diseases, and diabetes [34,35]. Vitamin B_2_ functions mainly as 2 coenzyme forms (flavin mononucleotide and flavin adenine dinucleotide) of redox enzymes. It is involved in the recycling of glutathione, which is an important antioxidant that protects against free radicals [36]. Previous research has indicated that a decrease in reduced glutathione levels occurs in response to vitamin B_2_ deficiency [37]. Therefore, vitamin B_2_ deficiency may be associated with the pathogenesis of CRC.

In our pre-matched analysis, although none of the associations between genetic polymorphisms and CRC risk were significant, in the dominant model, those who carry at least 1 minor allele (G) of the *MTRR* rs1801394 genetic polymorphism had a marginally significant correlation with CRC risk. However, in the matched population, we observed that those who carried at least 1 minor allele (G) of *MTRR* rs1801394 showed a significantly higher risk of CRC in the dominant model. A meta-analysis of the associations of the rs1801394 methionine synthase reductase polymorphism in CRC with a sample size of 20,945 revealed that there was a lack of evidence for an overall association between *MTRR* rs1801394 and CRC. However, they suggested ethnic-specific associations with Asian susceptibility and protection in comparisons of the A allele and A/G genotype, respectively [38]. Another study with evidence from 35 case-control studies did not find a significant association [39], but Zhou et al. [40] reported that the risk was increased for Caucasian patients who had at least 1 G allele, but not for Asians. However, we found that there was a synergistic effect on reducing CRC risk in males who carried the A allele and had a high vitamin B_2_ intake. A possible functional explanation for the biological plausibility of the *MTRR* rs1801394 A/A genotype preventing CRC risk could relate to its modulation of *MTR* activity, which may affect the levels of SAM and DNA methylation reactions [19]. A study conducted in Japan concluded that genetic polymorphisms of *MTRR* may interact with folate and vitamin B6 but not with vitamin B_2_ to increase CRC risk [41]. Le Marchand et al. [16] also reported that the *MTRR* rs1801394 polymorphism was associated with CRC risk but did not interact with folate, vitamin B_2_, vitamin B6, or vitamin B12. However, we found that *MTRR* rs1801394 could interact with vitamin B_2_ intake to reduce CRC risk, especially in males. There are variations in major and minor allele frequencies of both SNPs across different ethnicities. For instance, for the *MTRR* rs1801394 genetic polymorphism, in the European population, A= 0.52-0.55 and G = 0.45-0.48; in the Asian population, A = 0.68-0.75 and G= 0.29-0.32; and in Hawaiians, A= 0.68 and G= 0.32 [42]. For the *MTR* rs1805087 genetic polymorphism, in the European population, A= 0.82-0.84 and G= 0.16-0.18; in the Asian population, A= 0.72-0.82 and G= 0.18-0.28; and for the Hawaiian population, A= 0.86 and G= 0.14 [43]. Thus, the conflicting results observed for the association between vitamin B_2_ and CRC risk could be attributed to the fact that there are variations in major and minor allele frequencies across different ethnicities, which may lead to genetic heterogeneity. Although no exact mechanism has been identified as related to this interactive effect, it could be suggested that the protective effect of vitamin B_2_, which acts as a cofactor for MTHFR, which catalyzes the reduction of 5,10-methyl THF to 5-methyl THF for the latter DNA methylation reaction [6] could also modulate *MTR* to maintain the levels of SAM, which is involved in DNA methylation, while reducing the level of homocysteine to prevent colorectal carcinogenesis [8].

We found no significant association between the *MTR* rs1805087 genetic polymorphism and CRC risk. This is a common variant in the *MTR* gene, which consists of an A-to-G transition at base-pair 2756 and leads to a change from aspartic acid to glycine at codon 919 [44]. A meta-analysis investigated the relationship between *MTR* rs1805087 and CRC based on the findings of 27 studies and reported that this genetic polymorphism is not associated with CRC risk [45]. *MTR*, on chromosome 1q43, is responsible for encoding an enzyme involved in folate-mediated one-carbon metabolism, catalyzing the methylation of homocysteine to methionine with simultaneous conversion of 5-methyl-THF to THF [45]. It has been reported that *MTR* is necessary for the provision of SAM, which is a universal donor of methyl groups, and THF for nucleotide synthesis [46]. Although a direct function related to CRC has not been well characterized, individuals with the GG genotype may have lower homocysteine levels [47] and higher serum folate levels [48]. This may suggest that the G allele could be associated with beneficial effects to prevent carcinogenesis. Interestingly, we found an interaction between the *MTR* rs1805087 genetic polymorphism and vitamin B_2_ in males. Specifically, individuals who were homozygous for the major allele (A) and had a higher intake of vitamin B_2_ had a significantly reduced risk. A study investigated the interaction between vitamin B_2_ and the *MTR* rs1805087 genetic polymorphism and revealed that there was no significant interaction [49]. No significant associations were identified between the *MTR* rs1805087 polymorphism and CRC risk in the overall population or the Japanese population, with corresponding effect estimates of 1.1 and 1.0, respectively [16,50]. These results are consistent with our study findings in the Korean population, where the GG genotype was associated with a greater risk of CRC, with an OR of 1.75 and 1.85 for pre-matched and matched populations, respectively, but this difference was not significant. Since vitamin B_2_ also lowers homocysteine levels, it could be hypothesized that the interactive effect observed in the present study might be a biologically plausible approach for reducing CRC risk, especially in major allele carriers.

The major strength of the current study is the relatively large population in which gene‒diet interactions were observed with a case-control study design compared to previous case-control studies. Second, we used a validated SQFFQ, which was comprehensively designed to assess usual dietary intake among Koreans. Third, we performed gene-nutrient interaction analysis using pre-matched and matched populations to compare the results because propensity score matching can be used to control for confounding by making the exposed and unexposed groups as comparable as possible with respect to relevant confounding variables. Fourth, several potential confounding variables were considered; these variables were collected through a comprehensive questionnaire and a validated SQFFQ by well-trained interviewers. Despite the strengths of our study, several limitations need to be acknowledged. Although we focused on vitamin B_2_ since its interactive effect on CRC risk has not been well addressed in previous epidemiological evidence, several other vitamins, such as B6, B12, and folate, are essential for methylation reactions. Thus, multiple genes in related pathways other than *MTRR* and *MTR* could be involved. Healthy participants in this study were recruited from those who voluntarily participated in a health screening program in Korea. Thus, the control participants may have been relatively more concerned about health-related behaviors that might be associated with a reduced risk of CRC. Additionally, the recall of diet may have differed between cases and controls due to differences in health and behaviors. However, we tried to collect information on patients’ past habitual diet and lifestyle information prior to the diagnosis of cancer soon after their hospital admission, which might have reduced potential recall bias.

In conclusion, we found a significantly lower risk of CRC in patients who consume more vitamin B_2_. However, this association could vary depending on *MTRR* rs1801394 and *MTR* rs1805087 genetic polymorphisms. Specifically, the A/A genotype of *MTRR* rs1801394 combined with high vitamin B_2_ intake could have synergistic effects decreasing CRC risk in males, and homozygosity for the major allele (A) of the *MTR* rs1805087 polymorphism could interact with vitamin B_2_ to reduce CRC risk in males.

## Figures and Tables

**Figure f2-epih-46-e2024037:**
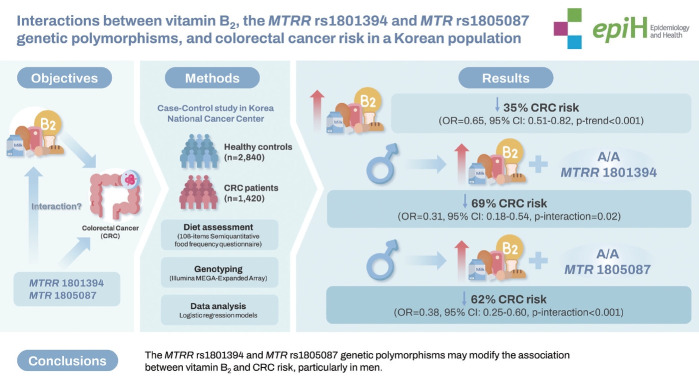


**Table 1.** General characteristics of the study population

Characteristics	Total (n=4,260)			Male (n=2,748)			Female (n=1,512)		
	Control (n=2,840)	Case (n=1,420)	p-value^1^	Control (n=1,832)	Case (n=916)	p-value^1^	Control (n=1,008)	Case (n=504)	p-value^1^
Age (yr)	57.61±9.48	58.13±10.25	0.110	57.90±9.06	58.51±9.95	0.120	57.08±10.18	57.44±10.76	0.530
Sex									
Male	1,832 (64.5)	916 (64.5)		-	-	-	-		
Female	1,008 (35.5)	504 (35.5)		-	-	-	-		
Body mass index (kg/m^2^)	24.06±2.83	24.08±3.41	0.870	24.44±2.78	24.09±3.11	0.004	23.36±2.80	24.06±3.90	<0.001
<18.5	38 (1.3)	48 (3.4)	0.003	15 (0.8)	27 (3.0)	<0.001	23 (2.3)	21 (4.2)	<0.001
18.5-<23.0	957 (33.7)	528 (37.2)		515 (28.1)	323 (35.3)		442 (43.9)	205 (40.7)	
23.0-<25.0	848 (30.0)	335 (23.6)		569 (31.1)	233 (25.4)		279 (27.7)	102 (20.2)	
≥25.0	942 (33.2)	509 (35.9)		707 (38.6)	333 (36.4)		235 (23.3)	176 (34.9)	
Missing	55 (1.9)	0 (0.0)		26 (1.4)	0 (0.0)		29 (2.9)	0 (0.0)	
Family history of CRC			<0.001			<0.001			0.230
Yes	135 (4.8)	122 (8.6)		78 (4.3)	83 (9.1)		57 (5.7)	39 (7.7)	
No	2,701 (95.1)	1,298 (91.4)		1,751 (95.6)	833 (90.9)		950 (94.3)	465 (92.3)	
Missing	4 (0.1)	0 (0.0)		3 (0.2)	0 (0.0)		1 (0.1)	0 (0.0)	
Supplement use			<0.001			<0.001			<0.001
Yes	2,116 (74.5)	789 (55.6)		1,315 (71.8)	501 (54.7)		801 (79.5)	288 (57.1)	
No	685 (24.1)	627 (44.2)		495 (27.0)	414 (45.2)		190 (18.9)	213 (42.3)	
Missing	39 (1.4)	4 (0.3)		22 (1.2)	1 (0.1)		17 (1.7)	3 (0.6)	
Marital status			0.009			0.180			0.020
Married	2,404 (84.7)	1,243 (87.5)		1,637 (89.4)	830 (90.6)		767 (76.1)	413 (81.9)	
Single	83 (2.9)	29 (2.0)		40 (2.2)	19 (2.1)		43 (4.3)	10 (2.0)	
Divorced, widowed, other	327 (11.5)	147 (10.4)		142 (7.8)	67 (7.3)		185 (18.4)	80 (15.9)	
Missing	26 (0.9)	1 (0.1)		13 (0.7)	0 (0.0)		13 (2.3)	1 (0.2)	
Education			<0.001			<0.001			<0.001
≤Elementary school	174 (6.1)	253 (17.8)		77 (4.2)	114 (12.5)		97 (9.6)	139 (27.6)	
Middle school	205 (7.2)	204 (14.4)		127 (6.9)	135 (14.7)		78 (7.7)	69 (13.7)	
High school	1,184 (41.7)	592 (41.7)		710 (38.8)	395 (43.1)		474 (47.0)	197 (39.1)	
≥College	1,236 (43.5)	369 (26.0)		886 (48.4)	272 (29.7)		350 (34.7)	97 (19.3)	
Missing	41 (1.4)	2 (0.1)		32 (1.8)	0 (0.0)		9 (0.9)	2 (0.4)	
Monthly income (10,000 KRW/mo)			<0.001			<0.001			<0.001
<200	652 (23.0)	561 (39.5)		365 (19.9)	359 (39.2)		287 (28.5)	202 (40.1)	
200-400	1,075 (37.9)	518 (36.5)		718 (39.2)	330 (36.0)		357 (35.4)	188 (37.3)	
≥400	1,025 (36.1)	328 (23.1)		693 (37.8)	220 (24.0)		332 (32.9)	108 (21.4)	
Missing	88 (3.1)	13 (0.9)		56 (3.1)	7 (0.8)		32 (3.2)	6 (1.2)	
Occupation			<0.001			<0.001			0.070
Housewife	582 (20.5)	303 (21.3)		5 (0.3)	1 (0.1)		577 (57.2)	302 (59.9)	
Professional, office worker	792 (27.9)	332 (23.4)		604 (33.0)	268 (29.3)		188 (18.7)	64 (12.7)	
Sales, service	574 (20.2)	90 (6.3)		437 (23.9)	60 (6.6)		137 (13.6)	30 (6.0)	
Agriculture, laborer, unemployed, other	862 (30.4)	694 (48.9)		764 (41.7)	587 (64.1)		98 (9.7)	107 (21.2)	
Missing	30 (1.1)	1 (0.1)		22 (1.2)	0 (0.0)		8 (0.8)	1 (0.2)	
Smoking status			0.230			0.020			0.070
Current	471 (16.6)	230 (16.2)		446 (24.3)	210 (22.9)		25 (2.5)	20 (4.0)	
Former	1,058 (37.3)	500 (35.2)		1,014 (55.4)	472 (51.5)		44 (4.4)	28 (5.6)	
Never	1,311 (46.2)	689 (48.5)		372 (20.3)	234 (25.6)		939 (93.2)	455 (90.3)	
Missing	0 (0.0)	1 (0.1)		0 (0.0)	0 (0.0)		0 (0.0)	1 (0.2)	
Alcohol consumption			<0.001			<0.001			<0.001
Current	1,710 (60.2)	685 (48.2)		1,299 (70.9)	551 (60.2)		411 (40.8)	134 (26.6)	
Former	272 (9.6)	204 (14.4)		227 (12.4)	161 (17.6)		45 (4.5)	43 (8.5)	
Never	858 (30.2)	530 (37.3)		306 (16.7)	204 (22.3)		552 (54.8)	326 (64.7)	
Missing	0 (0.0)	1 (0.1)		0 (0.0)	0 (0.0)		0 (0.0)	1 (0.2)	
Regular exercise			<0.001			<0.001			<0.001
Yes	1,589 (56.0)	504 (35.5)		1,070 (58.4)	344 (37.6)		519 (51.5)	160 (31.8)	
No	1,101 (38.8)	916 (64.5)		732 (40.0)	572 (62.5)		369 (36.6)	344 (68.3)	
Missing	150 (5.3)	0 (0.0)		30 (1.6)	0 (0.0)		120 (11.9)	0 (0.0)	
Physical activity (MET-min/wk)	2,627.4±2,781.1	2,145.9±1,999.8	<0.001	2,918.4±2,925.4	2,327.5±2,094.9	<0.001	2,098.8±2,410.7	1,816.5±1,769.6	0.010
Red meat intake (g/day)^2^	55.64±40.08	47.62±35.14	<0.001	57.94±42.09	50.82±37.35	<0.001	51.46±35.77	41.81±29.88	<0.001

Values are presented as mean±standard deviation or number (%). CRC, colorectal cancer; KRW, Korean won; MET, metabolic equivalent of task.

1 Using the chi-square and Student t-test for continuous and categorical variables, respectively.

2 Red meat intake was adjusted for total energy intake using residual method.

**Table 2.** Comparison of vitamin B2 intake between colorectal cancer cases and controls

Variables	Total			Male			Female		
	Controls (n=2,840)	Cases (n=1,420)	p-value^1^	Controls (n=1,832)	Cases (n=916)	p-value^1^	Controls (n=1,008)	Cases (n=504)	p-value^1^
Total energy intake (kcal/day)	1,741.1±567.0	2,043.6±575.2	<0.001	1,785.1±547.8	2,162.6±542.5	<0.001	1,661.1±592.4	1,827.3±570.4	<0.001
Vitamin B2 (mg/day)	1.21±0.38	1.12±0.33	<0.001	1.15±0.35	1.07±0.31	<0.001	1.32±0.41	1.19±0.35	<0.001

Values are presented as mean±standard deviation.

1 Using the Student t-test.

**Table 3.** Association between vitamin B2 intake and the risk of colorectal cancer (CRC)^1^

Vitamin B2	CRC				Proximal colon cancer			Distal colon cancer (mg/day)			Rectal cancer		
	Controls	Cases	Model I	Model II	Cases	Model I	Model II	Cases	Model I	Model II	Cases	Model I	Model II
Total													
Q1 (<0.94)	710 (25.0)	464 (32.7)	1.00 (reference)	1.00 (reference)	122 (27.7)	1.00 (reference)	1.00 (reference)	140 (30.8)	1.00 (reference)	1.00 (reference)	189 (37.4)	1.00 (reference)	1.00 (reference)
Q2 (0.94-1.17)	710 (25.0)	446 (31.4)	0.96 (0.81, 1.14)	1.01 (0.82, 1.23)	136 (30.9)	1.12 (0.86, 1.45)	1.15 (0.86, 1.53)	141 (31.1)	1.01 (0.78, 1.30)	1.04 (0.79, 1.38)	165 (32.7)	0.87 (0.69, 1.10)	0.94 (0.71, 1.23)
Q3 (1.17-1.43)	710 (25.0)	296 (20.9)	0.64 (0.53, 0.76)	0.69 (0.55, 0.86)	99 (22.5)	0.81 (0.61, 1.08)	0.84 (0.61, 1.15)	91 (20.0)	0.65 (0.49, 0.71)	0.66 (0.48, 0.91)	103 (20.4)	0.55 (0.42, 0.71)	0.63 (0.46, 0.85)
Q4 (≥1.43)	710 (25.0)	214 (15.1)	0.46 (0.38, 0.56)	0.65 (0.51, 0.82)	83 (18.9)	0.68 (0.51, 0.92)	0.87 (0.62, 1.23)	82 (18.1)	0.59 (0.44, 0.78)	0.76 (0.54, 1.06)	48 (9.5)	0.25 (0.18, 0.36)	0.40 (0.27, 0.58)
p for trend			<0.001	<0.001		0.002	0.193		<0.001	0.020		<0.001	<0.001
Male													
Q1 (<0.90)	458 (25.0)	278 (31.4)	1.00 (reference)	1.00 (reference)	62 (22.9)	1.00 (reference)	1.00 (reference)	79 (28.4)	1.00 (reference)	1.00 (reference)	129 (36.5)	1.00 (reference)	1.00 (reference)
Q2 (0.90-1.11)	458 (25.0)	312 (34.1)	1.12 (0.91, 1.38)	1.13 (0.88, 1.45)	95 (35.1)	1.53 (1.08, 2.16)	1.54 (1.06, 2.25)	99 (35.6)	1.25 (0.91, 1.73)	1.22 (0.86, 1.74)	115 (32.6)	0.89 (0.67, 1.18)	0.87 (0.63, 1.21)
Q3 (1.11-1.36)	458 (25.0)	185 (20.2)	0.67 (0.53, 0.84)	0.70 (0.53, 0.92)	57 (21.0)	0.92 (0.63, 1.35)	0.95 (0.63, 1.45)	55 (19.8)	0.70 (0.48, 1.01)	0.77 (0.45, 1.00)	71 (20.1)	0.55 (0.40, 0.76)	0.60 (0.41, 0.86)
Q4 (≥1.36)	458 (25.0)	141 (15.4)	0.51 (0.40, 0.65)	0.60 (0.45, 0.82)	57 (21.0)	0.92 (0.63, 1.35)	1.06 (0.68, 1.64)	45 (16.2)	0.57 (0.39, 0.84)	0.60 (0.39, 0.93)	38 (10.8)	0.30 (0.20, 0.43)	0.40 (0.23, 0.58)
p for trend			<0.001	<0.001		0.190	0.730		<0.001	0.008		<0.001	<0.001
Female													
Q1 (<1.03)	252 (25.0)	177 (35.1)	1.00 (reference)	1.00 (reference)	57 (33.7)	1.00 (reference)	1.00 (reference)	55 (31.3)	1.00 (reference)	1.00 (reference)	60 (39.5)	1.00 (reference)	1.00 (reference)
Q2 (1.03-1.30)	252 (25.0)	166 (32.9)	0.94 (0.71, 1.23)	0.98 (0.70, 1.38)	53 (31.4)	0.93 (0.62, 1.41)	0.98 (0.62, 1.56)	58 (33.0)	1.06 (0.70, 1.59)	1.11 (0.70, 1.78)	53 (34.9)	0.88 (0.59, 1.33)	0.90 (0.56, 1.44)
Q3 (1.30-1.60)	252 (25.0)	101 (20.0)	0.57 (0.42, 0.77)	0.79 (0.54, 1.14)	32 (18.9)	0.56 (0.35, 0.89)	0.78 (0.46, 1.31)	39 (22.2)	0.71 (0.45, 1.11)	0.97 (0.58, 1.63)	30 (19.7)	0.50 (0.31, 0.80)	0.67 (0.39, 1.16)
Q4 (≥1.60)	252 (25.0)	60 (11.9)	0.34 (0.24, 0.48)	0.60 (0.40, 0.90)	27 (16.0)	0.47 (0.29, 0.77)	0.71 (0.41, 1.24)	24 (13.6)	0.44 (0.26, 0.73)	0.80 (0.45, 1.44)	9 (5.9)	0.15 (0.07, 0.31)	0.27 (0.12, 0.60)
p for trend			<0.001	0.008		<0.001	0.146		<0.001	0.404		<0.001	<0.001

Values are presented as number (%) or odds ratio (95% confidence interval).

1 Model I: Crude model; Model II: Adjusted for age, sex, body mass index, alcohol consumption, smoking status, marital status, occupation, education, family history of CRC, supplement use, monthly income, regular exercise, red meat intake, and total energy intake; The sex variable was excluded for the male and female groups.

**Table 4.** Interaction between vitamin B2 intake and the MTRR rs1801394 and MTR rs1805087 genetic polymorphisms on colorectal cancer (CRC) risk in the matched population^1^

Vitamin B2 intake (mg/day)	A/A				G/A+G/G				p for interaction^2^
	Q1	Q2	Q3	Q4	Q1	Q2	Q3	Q4	
*MTRR* rs1801394									
All	<0.98	0.98-1.20	1.20-1.46	≥1.46	<0.98	0.98-1.20	1.20-1.46	≥1.46	
No. of controls/cases	145/200	146/177	138/103	147/79	125/185	124/153	133/98	123/86	
Model I	1.00 (reference)	0.86 (0.63, 1.18)	0.52 (0.37, 0.73)	0.37 (0.26, 0.53)	1.04 (0.76, 1.44)	0.86 (0.62, 1.18)	0.51 (0.36, 0.73)	0.47 (0.33, 0.68)	0.57
Model II	1.00 (reference)	0.56 (0.35, 0.88)	0.56 (0.33, 0.93)	0.72 (0.42, 1.22)	1.30 (0.82, 2.07)	1.49 (0.92, 2.40)	0.76 (0.45, 1.26)	1.05 (0.60, 1.83)	0.43
Male	<0.94	0.94-1.15	1.15-1.38	≥1.38	<0.94	0.94-1.15	1.15-1.38	≥1.38	
No. of controls/cases	83/132	97/111	94/70	99/48	86/112	73/102	75/56	70/60	
Model I	1.00 (reference)	0.72 (0.49, 1.06)	0.47 (0.31, 0.71)	0.31 (0.20, 0.47)	0.82 (0.55, 1.21)	0.88 (0.59, 1.32)	0.47 (0.30, 0.73)	0.54 (0.35, 0.84)	0.04
Model II	1.00 (reference)	0.57 (0.36, 0.92)	0.47 (0.29, 0.79)	0.31 (0.18, 0.54)	0.73 (0.45, 1.18)	0.87 (0.52, 1.44)	0.51 (0.30, 0.86)	0.62 (0.36, 1.08)	0.02
Female	<1.05	1.05-1.32	1.32-1.61	≥1.61	<1.05	1.05-1.32	1.32-1.61	≥1.61	
No. of controls/cases	60/75	50/61	42/40	51/22	41/68	51/66	59/32	50/26	
Model I	1.00 (reference)	0.98 (0.59, 1.62)	0.76 (0.44, 1.32)	0.35 (0.19, 0.63)	1.33 (0.79, 2.22)	1.04 (0.63, 1.71)	0.43 (0.25, 0.75)	0.42 (0.23, 0.75)	0.62
Model II	1.00 (reference)	1.57 (0.83, 2.98)	2.00 (0.96, 4.15)	1.05 (0.48, 2.34)	1.49 (0.77, 2.89)	1.86 (0.99, 3.52)	1.13 (0.56, 2.28)	1.21 (0.57, 2.55)	0.44
*MTR* rs1805087									
All	<0.98	0.98-1.20	1.20-1.46	≥1.46	<0.98	0.98-1.20	1.20-1.46	≥1.46	
No. of controls/cases	192/286	199/255	204/140	219/126	78/99	71/75	67/61	51/39	
Model I	1.00 (reference)	0.86 (0.66, 1.12)	0.46 (0.34, 0.60)	0.37 (0.27, 0.50)	0.86 (0.61, 1.22)	0.68 (0.47, 0.99)	0.61 (0.41, 0.91)	0.48 (0.30, 0.78)	0.12
Model II	1.00 (reference)	0.74 (0.50, 1.08)	0.42 (0.27, 0.66)	0.72 (0.46, 1.13)	0.78 (0.45, 1.35)	0.54 (0.30, 0.98)	0.93 (0.52, 1.65)	0.78 (0.37, 1.65)	0.42
Male	<0.94	0.94-1.15	1.15-1.38	≥1.38	<0.94	0.94-1.15	1.15-1.38	≥1.38	
No. of controls/cases	115/184	121/161	133/85	142/85	54/60	49/52	36/41	27/23	
Model I	1.00 (reference)	0.83 (0.60, 1.16)	0.40 (0.28, 0.57)	0.37 (0.26, 0.53)	0.69 (0.45, 1.07)	0.66 (0.42, 1.05)	0.71 (0.43, 1.17)	0.53 (0.29, 0.97)	0.02
Model II	1.00 (reference)	0.75 (0.50, 1.13)	0.38 (0.25, 0.60)	0.39 (0.25, 0.60)	0.52 (0.30, 0.87)	0.47 (0.27, 0.83)	0.82 (0.45, 1.50)	0.65 (0.30, 1.40)	<0.001
Female	<1.05	1.05-1.32	1.32-1.61	≥1.61	<1.05	1.05-1.32	1.32-1.61	≥1.61	
No. of controls/cases	79/106	73/95	70/57	81/34	22/37	28/32	31/15	20/14	
Model I	1.00 (reference)	0.97 (0.64, 1.48)	0.61 (0.39, 0.96)	0.31 (0.19, 0.51)	1.25 (0.69, 2.29)	0.85 (0.48, 1.53)	0.36 (0.18, 0.71)	0.52 (0.25, 1.10)	0.94
Model II	1.00 (reference)	1.69 (0.98, 2.91)	1.61 (0.87, 2.97)	0.87 (0.46, 1.67)	1.21 (0.57, 2.57)	1.12 (0.54, 2.33)	0.82 (0.36, 1.87)	1.44 (0.56, 3.74)	>0.99

Values are presented as odds ratio (95% confidence interval).

1 Model I: Crude model; Model II: Adjusted for age, sex, body mass index, alcohol consumption, smoking status, marital status, occupation, education, family history of CRC, supplement use, monthly income, regular exercise, red meat intake, and total energy intake; The sex variable was excluded for the male and female groups.

2 False discovery rate–adjusted p-values.

## Supplementary materials

Supplementary Material 1. Table. General characteristics of the population related to gene-nutrient interaction in pre-matched population

Supplementary Material 2. General characteristics of the population related to gene-nutrient interaction in matched population

Supplementary Material 3. Association between the *MTRR* rs1801394 and *MTR* rs1805087 genetic polymorphisms and CRC risk in the pre-matched population

Supplementary Material 4. Association between the *MTRR* rs1801394 (A66G) and *MTR* rs1805087 (A2756G) genetic polymorphisms and CRC risk in the matched population

Supplementary Material 5. . Interaction between vitamin B_2_ intake and the *MTRR* A66G and *MTR* A2756G genetic polymorphisms on CRC risk in the pre-matched population.

## References

[1] Sung H, Ferlay J, Siegel RL, Laversanne M, Soerjomataram I, Jemal A (2021). Global cancer statistics 2020: GLOBOCAN estimates of incidence and mortality worldwide for 36 cancers in 185 countries. CA Cancer J Clin.

[2] Kang MJ, Won YJ, Lee JJ, Jung KW, Kim HJ, Kong HJ (2022). Cancer statistics in Korea: incidence, mortality, survival, and prevalence in 2019. Cancer Res Treat.

[3] Sninsky JA, Shore BM, Lupu GV, Crockett SD (2022). Risk factors for colorectal polyps and cancer. Gastrointest Endosc Clin N Am.

[4] World Cancer Research Fund/American Institute for Cancer Research Diet, nutrition, physical activity, and cancer: a global perspective. A summary of the third expert report.

[5] Arigony AL, de Oliveira IM, Machado M, Bordin DL, Bergter L, Prá D (2013). The influence of micronutrients in cell culture: a reflection on viability and genomic stability. Biomed Res Int.

[6] de Vogel S, Dindore V, van Engeland M, Goldbohm RA, van den Brandt PA, Weijenberg MP (2008). Dietary folate, methionine, riboflavin, and vitamin B-6 and risk of sporadic colorectal cancer. J Nutr.

[7] McNulty H, Scott JM (2008). Intake and status of folate and related Bvitamins: considerations and challenges in achieving optimal status. Br J Nutr.

[8] Jacques PF, Kalmbach R, Bagley PJ, Russo GT, Rogers G, Wilson PW (2002). The relationship between riboflavin and plasma total homocysteine in the Framingham Offspring cohort is influenced by folate status and the C677T transition in the methylenetetrahydrofolate reductase gene. J Nutr.

[9] Wu LL, Wu JT (2002). Hyperhomocysteinemia is a risk factor for cancer and a new potential tumor marker. Clin Chim Acta.

[10] Sun CF, Haven TR, Wu TL, Tsao KC, Wu JT (2002). Serum total homocysteine increases with the rapid proliferation rate of tumor cells and decline upon cell death: a potential new tumor marker. Clin Chim Acta.

[11] Keshteli AH, Baracos VE, Madsen KL (2015). Hyperhomocysteinemia as a potential contributor of colorectal cancer development in inflammatory bowel diseases: a review. World J Gastroenterol.

[12] Battistelli S, Vittoria A, Stefanoni M, Bing C, Roviello F (2006). Total plasma homocysteine and methylenetetrahydrofolate reductase C677T polymorphism in patients with colorectal carcinoma. World J Gastroenterol.

[13] Lim YJ, Kim JH, Park SK, Son HJ, Kim JJ, Kim YH (2012). Hyperhomocysteinemia is a risk factor for colorectal adenoma in women. J Clin Biochem Nutr.

[14] Zschäbitz S, Cheng TY, Neuhouser ML, Zheng Y, Ray RM, Miller JW (2013). B vitamin intakes and incidence of colorectal cancer: results from the Women’s Health Initiative Observational Study cohort. Am J Clin Nutr.

[15] Sun Z, Zhu Y, Wang PP, Roebothan B, Zhao J, Zhao J (2012). Reported intake of selected micronutrients and risk of colorectal cancer: results from a large population-based case-control study in Newfoundland, Labrador and Ontario, Canada. Anticancer Res.

[16] Le Marchand L, Donlon T, Hankin JH, Kolonel LN, Wilkens LR, Seifried A (2002). B-vitamin intake, metabolic genes, and colorectal cancer risk (United States). Cancer Causes Control.

[17] Kabat GC, Miller AB, Jain M, Rohan TE (2008). Dietary intake of selected B vitamins in relation to risk of major cancers in women. Br J Cancer.

[18] Wu MH, Chen CH, Chen CP, Huang TL, Yueh TC, Wang ZH (2022). Contribution of 5-methyltetrahydrofolate-homocysteine methyltransferase reductase genotypes to colorectal cancer in Taiwan. Anticancer Res.

[19] Jokić M, Brčić-Kostić K, Stefulj J, Catela Ivković T, Božo L, Gamulin M (2011). Association of MTHFR, MTR, MTRR, RFC1, and DHFR gene polymorphisms with susceptibility to sporadic colon cancer. DNA Cell Biol.

[20] Pardini B, Kumar R, Naccarati A, Prasad RB, Forsti A, Polakova V (2011). MTHFR and MTRR genotype and haplotype analysis and colorectal cancer susceptibility in a case-control study from the Czech Republic. Mutat Res.

[21] Wettergren Y, Odin E, Carlsson G, Gustavsson B (2010). MTHFR, MTR, and MTRR polymorphisms in relation to p16INK4A hypermethylation in mucosa of patients with colorectal cancer. Mol Med.

[22] de Vogel S, Wouters KA, Gottschalk RW, van Schooten FJ, de Goeij AF, de Bruïne AP (2009). Genetic variants of methyl metabolizing enzymes and epigenetic regulators: associations with promoter CpG island hypermethylation in colorectal cancer. Cancer Epidemiol Biomarkers Prev.

[23] Ho D, Imai K, King G, Stuart EA (2011). MatchIt: nonparametric preprocessing for parametric causal inference. J Stat Softw.

[24] Ahn Y, Kwon E, Shim JE, Park MK, Joo Y, Kimm K (2007). Validation and reproducibility of food frequency questionnaire for Korean genome epidemiologic study. Eur J Clin Nutr.

[25] Eussen SJ, Vollset SE, Hustad S, Midttun Ø, Meyer K, Fredriksen A (2010). Plasma vitamins B_2_, B6, and B12, and related genetic variants as predictors of colorectal cancer risk. Cancer Epidemiol Biomarkers Prev.

[26] Yoon YS, Jung S, Zhang X, Ogino S, Giovannucci EL, Cho E (2016). Vitamin B_2_ intake and colorectal cancer risk; results from the Nurses’ Health Study and the Health Professionals Follow-Up Study cohort. Int J Cancer.

[27] Huang CY, Abulimiti A, Zhang X, Feng XL, Luo H, Chen YM (2020). Dietary B vitamin and methionine intakes and risk for colorectal cancer: a case-control study in China. Br J Nutr.

[28] Kim J, Lee J, Oh JH, Sohn DK, Shin A, Kim J (2022). Dietary methyl donor nutrients, DNA mismatch repair polymorphisms, and risk of colorectal cancer based on microsatellite instability status. Eur J Nutr.

[29] Sharp L, Little J, Brockton NT, Cotton SC, Masson LF, Haites NE (2008). Polymorphisms in the methylenetetrahydrofolate reductase (MTHFR) gene, intakes of folate and related B vitamins and colorectal cancer: a case-control study in a population with relatively low folate intake. Br J Nutr.

[30] Rivlin RS (1970). Riboflavin metabolism. N Engl J Med.

[31] Moat SJ, Ashfield-Watt PA, Powers HJ, Newcombe RG, McDowell IF (2003). Effect of riboflavin status on the homocysteine-lowering effect of folate in relation to the MTHFR (C677T) genotype. Clin Chem.

[32] Powers HJ (2003). Riboflavin (vitamin B-2) and health. Am J Clin Nutr.

[33] Webster RP, Gawde MD, Bhattacharya RK (1996). Modulation of carcinogen-induced DNA damage and repair enzyme activity by dietary riboflavin. Cancer Lett.

[34] Preiser JC (2012). Oxidative stress. JPEN J Parenter Enteral Nutr.

[35] Ashoori M, Saedisomeolia A (2014). Riboflavin (vitamin B₂) and oxidative stress: a review. Br J Nutr.

[36] Werner R, Manthey KC, Griffin JB, Zempleni J (2005). HepG2 cells develop signs of riboflavin deficiency within 4 days of culture in riboflavin-deficient medium. J Nutr Biochem.

[37] Wu G, Fang YZ, Yang S, Lupton JR, Turner ND (2004). Glutathione metabolism and its implications for health. J Nutr.

[38] Pabalan N, Singian E, Tabangay L, Jarjanazi H, Singh N (2015). Associations of the A66G methionine synthase reductase polymorphism in colorectal cancer: a systematic review and meta-analysis. Biomark Cancer.

[39] Han D, Shen C, Meng X, Bai J, Chen F, Yu Y (2012). Methionine synthase reductase A66G polymorphism contributes to tumor susceptibility: evidence from 35 case-control studies. Mol Biol Rep.

[40] Zhou D, Mei Q, Luo H, Tang B, Yu P (2012). The polymorphisms in methylenetetrahydrofolate reductase, methionine synthase, methionine synthase reductase, and the risk of colorectal cancer. Int J Biol Sci.

[41] Otani T, Iwasaki M, Hanaoka T, Kobayashi M, Ishihara J, Natsukawa S (2005). Folate, vitamin B6, vitamin B12, and vitamin B_2_ intake, genetic polymorphisms of related enzymes, and risk of colorectal cancer in a hospital-based case-control study in Japan. Nutr Cancer.

[42] Wu PP, Tang RN, An L (2015). A meta-analysis of MTRR A66G polymorphism and colorectal cancer susceptibility. J BUON.

[43] Yu K, Zhang J, Zhang J, Dou C, Gu S, Xie Y (2010). Methionine synthase A2756G polymorphism and cancer risk: a meta-analysis. Eur J Hum Genet.

[44] Chen J, Giovannucci E, Kelsey K, Rimm EB, Stampfer MJ, Colditz GA (1996). A methylenetetrahydrofolate reductase polymorphism and the risk of colorectal cancer. Cancer Res.

[45] Ding W, Zhou DL, Jiang X, Lu LS (2013). Methionine synthase A2756G polymorphism and risk of colorectal adenoma and cancer: evidence based on 27 studies. PLoS One.

[46] Banerjee RV, Matthews RG (1990). Cobalamin-dependent methionine synthase. FASEB J.

[47] Dekou V, Gudnason V, Hawe E, Miller GJ, Stansbie D, Humphries SE (2001). Gene-environment and gene-gene interaction in the determination of plasma homocysteine levels in healthy middle-aged men. Thromb Haemost.

[48] Chen J, Stampfer MJ, Ma J, Selhub J, Malinow MR, Hennekens CH (2001). Influence of a methionine synthase (D919G) polymorphism on plasma homocysteine and folate levels and relation to risk of myocardial infarction. Atherosclerosis.

[49] de Vogel S, Wouters KA, Gottschalk RW, van Schooten FJ, de Goeij AF, de Bruïne AP (2011). Dietary methyl donors, methyl metabolizing enzymes, and epigenetic regulators: diet-gene interactions and promoter CpG island hypermethylation in colorectal cancer. Cancer Causes Control.

[50] Sharp L, Little J (2004). Polymorphisms in genes involved in folate metabolism and colorectal neoplasia: a HuGE review. Am J Epidemiol.

