# Supplementary material for: Interactions between vitamin B2, the MTRR rs1801394 and MTR rs1805087 genetic polymorphisms, and colorectal cancer risk in a Korean population
Source: Epidemiol Health. 2024 Mar 11;46:e2024037. doi: 10.4178/epih.e2024037 (PMC11369566; doi:10.4178/epih.e2024037)
Supplement: Supplementary Material 1. — Table. General characteristics of the population related to gene-nutrient interaction in pre-matched population [file epih-46-e2024037-Supplementary-1.docx]

**Supplementary Tables**

Supplementary Material 1. Table. General characteristics of the population related to gene-nutrient interaction in pre-matched population

| Variables | Total (n=3,106) | |  |  | Male (n=1,923) | |  |  | Female (n=1,183) | |  |
| --- | --- | --- | --- | --- | --- | --- | --- | --- | --- | --- | --- |
|  | Control (n=2,025) | Case (n=1,081) | p-value^*^ |  | Control (n=1,232) | Case (n=691) | p-value^*^ |  | Control (n=793) | Case (n=390) | p-value^*^ |
| Age (years) | 55.57±8.97 | 58.28±10.27 | <0.001 |  | 55.64±8.65 | 58.60±10.08 | <0.001 |  | 55.46±9.44 | 57.72±10.59 | <0.001 |
| Sex |  |  | 0.091 |  |  |  |  |  |  |  |  |
| Male | 1232 (60.8) | 691 (63.9) |  |  |  |  |  |  |  |  |  |
| Female | 793 (39.2) | 390 (36.1) |  |  |  |  |  |  |  |  |  |
| Body mass index (kg/m^2^) | 24.06±2.88 | 24.17±3.47 | 0.350 |  | 24.54±2.84 | 24.28±3.20 | 0.071 |  | 23.30±2.78 | 23.99±3.89 | 0.002 |
| <18.5 | 30 (1.5) | 34 (3.2) | <0.001 |  | 8 (0.7) | 19 (2.8) | <0.001 |  | 22 (2.8) | 15 (3.9) | <0.001 |
| 18.5 - <23 | 688 (34.0) | 391 (36.2) |  |  | 334 (27.1) | 230 (33.3) |  |  | 354 (44.6) | 161 (41.3) |  |
| 23 - <25 | 583 (28.8) | 256 (23.7) |  |  | 375 (30.4) | 174 (25.2) |  |  | 208 (26.2) | 82 (21.0) |  |
| ≥25 | 682 (33.7) | 400 (37.0) |  |  | 496 (40.3) | 268 (38.8) |  |  | 186 (23.5) | 132 (33.9) |  |
| Missing | 42 (2.1) | 0 (0.0) |  |  | 19 (1.5) | 0 (0.0) |  |  | 23 (2.9) | 0 (0.0) |  |
| Family history of CRC |  |  | <0.001 |  |  |  | <0.001 |  |  |  | 0.295 |
| Yes | 97 (4.8) | 85 (7.9) |  |  | 52 (4.2) | 56 (8.1) |  |  | 45 (5.7) | 29 (7.4) |  |
| No | 1928 (95.2) | 996 (92.1) |  |  | 1180 (95.8) | 635 (91.9) |  |  | 748 (94.3) | 361 (92.6) |  |
| Missing |  | 0 (0.0) |  |  | 0 (0.0) | 0 (0.0) |  |  | 0 (0.0) | 0 (0.0) |  |
| Supplement use |  |  | <0.001 |  |  |  | <0.001 |  |  |  | <0.001 |
| Yes | 1471 (72.6) | 624 (57.7) |  |  | 857 (69.6) | 396 (57.3) |  |  | 614 (77.4) | 228 (58.5) |  |
| No | 527 (26.0) | 453 (41.9) |  |  | 362 (29.4) | 294 (42.6) |  |  | 165 (20.8) | 159 (40.8) |  |
| Missing | 27 (1.3) | 4 (0.4) |  |  | 13 (1.1) | 1 (0.1) |  |  | 14 (1.8) | 3 (0.8) |  |
| Marital status |  |  | 0.030 |  |  |  | 0.831 |  |  |  | 0.016 |
| Married | 1740 (85.9) | 960 (88.8) |  |  | 1122 (91.1) | 630 (91.2) |  |  | 618 (77.9) | 330 (84.6) |  |
| Single | 57 (2.8) | 19 (1.8) |  |  | 24 (2.0) | 13 (1.9) |  |  | 33 (4.2) | 6 (1.5) |  |
| Divorced, widowed, other | 215 (10.6) | 101 (9.3) |  |  | 81 (6.6) | 48 (7.0) |  |  | 134 (16.9) | 53 (13.6) |  |
| Missing | 13 (0.6) | 1 (0.1) |  |  | 5 (0.4) | 0 (0.0) |  |  | 8 (1.0) | 1 (0.3) |  |
| Education |  |  | <0.001 |  |  |  | <0.001 |  |  |  | <0.001 |
| ≤Elementary school | 112 (5.5) | 191 (17.7) |  |  | 47 (3.8) | 90 (13.0) |  |  | 65 (8.2) | 101 (25.9) |  |
| Middle school | 133 (6.6) | 163 (15.1) |  |  | 76 (6.2) | 103 (14.9) |  |  | 57 (7.2) | 60 (15.4) |  |
| High school | 857 (42.3) | 442 (40.9) |  |  | 487 (39.5) | 292 (42.3) |  |  | 370 (46.7) | 150 (38.5) |  |
| ≥College | 910 (44.9) | 283 (26.2) |  |  | 614 (49.8) | 206 (29.8) |  |  | 296 (37.3) | 77 (19.7) |  |
| Missing | 13 (0.6) | 2 (0.2) |  |  | 8 (0.7) | 0 (0.0) |  |  | 5 (0.6) | 2 (0.5) |  |
| Monthly income (10,000 Korean won/month) |  |  | <0.001 |  |  |  | <0.001 |  |  |  | <0.001 |
| <200 | 421 (20.8) | 440 (40.7) |  |  | 219 (17.8) | 281 (40.7) |  |  | 202 (25.5) | 159 (40.8) |  |
| 200-400 | 778 (38.4) | 392 (36.3) |  |  | 491 (39.9) | 248 (35.9) |  |  | 287 (36.2) | 144 (36.9) |  |
| ≥400 | 798 (39.4) | 239 (22.1) |  |  | 509 (41.3) | 155 (22.4) |  |  | 289 (36.4) | 84 (21.5) |  |
| Missing | 28 (1.4) | 10 (0.9) |  |  | 13 (1.1) | 7 (1.0) |  |  | 15 (2.0) | 3 (0.8) |  |
| Occupation |  |  | <0.001 |  |  |  | <0.001 |  |  |  | <0.001 |
| Housewife | 434 (21.4) | 231 (21.4) |  |  | 4 (0.3) | 1 (0.1) |  |  | 430 (54.2) | 230 (59.0) |  |
| Professional, office worker | 624 (30.8) | 263 (24.3) |  |  | 462 (37.5) | 211 (30.5) |  |  | 162 (20.4) | 52 (13.3) |  |
| Sales, service | 451 (22.3) | 79 (7.3) |  |  | 330 (26.8) | 51 (7.4) |  |  | 121 (15.3) | 28 (7.2) |  |
| Agriculture, laborer, unemployed, other | 503 (24.8) | 507 (46.9) |  |  | 429 (34.8) | 428 (61.9) |  |  | 74 (9.3) | 79 (20.3) |  |
| Missing | 13 (0.6) | 1 (0.1) |  |  | 7 (0.6) | 0 (0.0) |  |  | 6 (0.8) | 1 (0.3) |  |
| Smoking status |  |  | 0.027 |  |  |  | <0.001 |  |  |  | 0.397 |
| Current | 363 (17.9) | 154 (14.3) |  |  | 342 (27.8) | 140 (20.3) |  |  | 21 (2.7) | 14 (3.6) |  |
| Former | 704 (34.8) | 386 (35.7) |  |  | 663 (53.8) | 364 (52.7) |  |  | 41 (5.2) | 22 (5.6) |  |
| Never | 958 (47.3) | 540 (50.0) |  |  | 227 (18.4) | 187 (27.1) |  |  | 731 (92.2) | 353 (90.5) |  |
| Missing | 0 (0.0) | 1 (0.1) |  |  |  | 0 (0.0) |  |  | 0 (0.0) | 1 (0.3) |  |
| Alcohol consumption |  |  | <0.001 |  |  |  | <0.001 |  |  |  | <0.001 |
| Current | 1246 (61.5) | 505 (46.7) |  |  | 888 (72.1) | 403 (58.3) |  |  | 358 (45.2) | 102 (26.2) |  |
| Former | 184 (9.1) | 163 (15.1) |  |  | 146 (11.9) | 123 (17.8) |  |  | 38 (4.8) | 40 (10.3) |  |
| Never | 595 (29.4) | 412 (38.1) |  |  | 198 (16.1) | 165 (23.9) |  |  | 397 (50.1) | 247 (63.3) |  |
| Missing | 0 (0.0) | 1 (0.1) |  |  | 0 (0.0) | 0 (0.0) |  |  | 0 (0.0) | 1 (0.3) |  |
| Regular exercise |  |  | <0.001 |  |  |  | <0.001 |  |  |  | <0.001 |
| Yes | 1085 (53.6) | 387 (35.8) |  |  | 685 (55.6) | 254 (36.8) |  |  | 400 (50.4) | 133 (34.1) |  |
| No | 823 (40.6) | 694 (64.2) |  |  | 532 (43.2) | 437 (63.2) |  |  | 291 (36.7) | 257 (65.9) |  |
| Missing | 117 (5.8) | 0 (0.0) |  |  | 15 (1.2) | 0 (0.0) |  |  | 102 (12.9) | 0 (0.0) |  |
| Physical activity (MET-min/week) | 2540.7±2770.4 | 2124.3±2041.4 | <0.001 |  | 2816.3±2960.5 | 2290.4±2130.7 | <0.001 |  | 2112.7±2385.4 | 1830.9±1839.6 | 0.026 |
| Red meat intake (g/day)^†^ | 57.27±40.62 | 48.08±35.74 | <0.001 |  | 60.04±43.6 | 51.37±38.18 | <0.001 |  | 52.97±35.17 | 42.25±30.14 | <0.001 |

^*^p-value was obtained using the chi-square and student t-test for continuous and categorical variables, respectively.

^†^Red meat intake was adjusted for total energy intake using residual method.

CRC, colorectal cancer; MET, metabolic equivalent of task.
