# Supplementary material for: Interactions between vitamin B2, the MTRR rs1801394 and MTR rs1805087 genetic polymorphisms, and colorectal cancer risk in a Korean population
Source: Epidemiol Health. 2024 Mar 11;46:e2024037. doi: 10.4178/epih.e2024037 (PMC11369566; doi:10.4178/epih.e2024037)
Supplement: Supplementary Material 2. — General characteristics of the population related to gene-nutrient interaction in matched population [file epih-46-e2024037-Supplementary-2.docx]

Supplementary Material 2. General characteristics of the population related to gene-nutrient interaction in matched population

| Variables | Total (n=2162) | |  |  | Men (n=1368) | |  |  | Women (n=794) | |  |
| --- | --- | --- | --- | --- | --- | --- | --- | --- | --- | --- | --- |
|  | Control (n=1081) | Case (n=1081) | p-value^*^ |  | Control (n=677) | Case (n=691) | p-value^*^ |  | Control (n=404) | Case (n=390) | p-value^*^ |
| Age (years) | 57.93±9.69 | 58.28±10.27 | 0.41 |  | 58.12±9.28 | 58.60±10.08 | 0.37 |  | 57.61±10.35 | 57.72±10.59 | 0.88 |
| Sex |  |  | 0.53 |  |  |  |  |  |  |  |  |
| Male | 677 (62.6) | 691 (63.9) |  |  |  |  |  |  |  |  |  |
| Female | 404 (37.4) | 390 (36.1) |  |  |  |  |  |  |  |  |  |
| Body mass index (kg/m^2^) | 24.09±3.02 | 24.17±3.47 | 0.57 |  | 24.57±3.00 | 24.28±3.20 | 0.09 |  | 23.30±2.89 | 23.99±3.89 | 0.005 |
| <18.5 | 21 (1.9) | 34 (3.2) | 0.03 |  | 4 (0.6) | 19 (2.8) | <0.001 |  | 17 (4.2) | 15 (3.9) | 0.40 |
| 18.5 - <23 | 344 (31.8) | 391 (36.2) |  |  | 176 (26.0) | 230 (33.3) |  |  | 168 (41.6) | 161 (41.3) |  |
| 23 - <25 | 323 (29.9) | 256 (23.7) |  |  | 212 (31.3) | 174 (25.2) |  |  | 111 (27.5) | 82 (21.0) |  |
| ≥25 | 368 (34.0) | 400 (37.0) |  |  | 270 (39.9) | 268 (38.8) |  |  | 98 (24.3) | 132 (33.9) |  |
| Missing | 25 (2.3) | 0 (0.0) |  |  | 15 (2.2) | 0 (0.0) |  |  | 10 (2.5) | 0 (0.0) |  |
| Family history of CRC |  |  | 0.009 |  |  |  | 0.01 |  |  |  | 0.32 |
| Yes | 55 (5.1) | 85 (7.9) |  |  | 32 (4.7) | 56 (8.1) |  |  | 23 (5.7) | 29 (7.4) |  |
| No | 1026 (94.9) | 996 (92.1) |  |  | 645 (95.3) | 635 (91.9) |  |  | 381 (94.3) | 361 (92.6) |  |
| Missing | 0 (0.0) | 0 (0.0) |  |  | 0 (0.0) | 0 (0.0) |  |  | 0 (0.0) | 0 (0.0) |  |
| Supplement use |  |  | <0.001 |  |  |  | <0.001 |  |  |  | <0.001 |
| Yes | 819 (75.8) | 624 (57.7) |  |  | 496 (73.3) | 396 (57.3) |  |  | 323 (80.0) | 228 (58.5) |  |
| No | 249 (23.0) | 453 (41.9) |  |  | 173 (25.5) | 294 (42.6) |  |  | 76 (18.8) | 159 (40.8) |  |
| Missing | 13 (1.2) | 4 (0.4) |  |  | 8 (1.2) | 1 (0.1) |  |  | 5 (1.2) | 3 (0.8) |  |
| Marital status |  |  | 0.03 |  |  |  | 0.68 |  |  |  | 0.01 |
| Married | 929 (85.9) | 960 (88.8) |  |  | 617 (91.1) | 630 (91.2) |  |  | 312 (77.2) | 330 (84.6) |  |
| Single | 26 (2.4) | 19 (1.8) |  |  | 10 (1.5) | 13 (1.9) |  |  | 16 (4.0) | 6 (1.5) |  |
| Divorced, widowed, other | 116 (10.7) | 101 (9.3) |  |  | 45 (6.7) | 48 (7.0) |  |  | 71 (17.6) | 53 (13.6) |  |
| Missing | 10 (0.9) | 1 (0.1) |  |  | 5 (0.7) | 0 (0.0) |  |  | 5 (1.2) | 1 (0.3) |  |
| Education |  |  | <0.001 |  |  |  | <0.001 |  |  |  | <0.001 |
| ≤Elementary school | 64 (5.9) | 191 (17.7) |  |  | 30 (4.4) | 90 (13.0) |  |  | 34 (8.4) | 101 (25.9) |  |
| Middle school | 76 (7.0) | 163 (15.1) |  |  | 46 (6.8) | 103 (14.9) |  |  | 30 (7.4) | 60 (15.4) |  |
| High school | 432 (40.0) | 442 (40.9) |  |  | 252 (37.2) | 292 (42.3) |  |  | 180 (44.6) | 150 (38.5) |  |
| ≥College | 496 (45.9) | 283 (26.2) |  |  | 341 (50.4) | 206 (29.8) |  |  | 155 (38.4) | 77 (19.7) |  |
| Missing | 13 (1.2) | 2 (0.2) |  |  | 8 (1.2) | 0 (0.0) |  |  | 5 (1.2) | 2 (0.5) |  |
| Monthly income (10,000 Korean won/month) |  |  | <0.001 |  |  |  | <0.001 |  |  |  | <0.001 |
| <200 | 236 (21.8) | 440 (40.7) |  |  | 137 (20.2) | 281 (40.7) |  |  | 99 (24.5) | 159 (40.8) |  |
| 200-400 | 413 (38.2) | 392 (36.3) |  |  | 264 (39.0) | 248 (35.9) |  |  | 149 (36.9) | 144 (36.9) |  |
| ≥400 | 405 (37.5) | 239 (22.1) |  |  | 264 (39.0) | 155 (22.4) |  |  | 141 (34.9) | 84 (21.5) |  |
| Missing | 27 (2.5) | 10 (0.9) |  |  | 12 (1.8) | 7 (1.0) |  |  | 15 (3.7) | 3 (0.8) |  |
| Occupation |  |  | <0.001 |  |  |  | <0.001 |  |  |  | 0.24 |
| Housewife | 228 (21.1) | 231 (21.4) |  |  | 3 (0.4) | 1 (0.1) |  |  | 225 (55.7) | 230 (59.0) |  |
| Professional, office worker | 314 (29.1) | 263 (24.3) |  |  | 228 (33.7) | 211 (30.5) |  |  | 86 (21.3) | 52 (13.3) |  |
| Sales, service | 218 (20.2) | 79 (7.3) |  |  | 170 (25.1) | 51 (7.4) |  |  | 48 (11.9) | 28 (7.2) |  |
| Agriculture, laborer, unemployed, other | 308 (28.5) | 507 (46.9) |  |  | 269 (39.7) | 428 (61.9) |  |  | 39 (9.7) | 79 (20.3) |  |
| Missing | 13 (1.2) | 1 (0.1) |  |  | 7 (1.0) | 0 (0.0) |  |  | 6 (1.5) | 1 (0.3) |  |
| Smoking status |  |  | 0.16 |  |  |  | 0.002 |  |  |  | 0.11 |
| Current | 170 (15.7) | 154 (14.3) |  |  | 163 (24.1) | 140 (20.3) |  |  | 7 (1.7) | 14 (3.6) |  |
| Former | 400 (37.0) | 386 (35.7) |  |  | 381 (56.3) | 364 (52.7) |  |  | 19 (4.7) | 22 (5.6) |  |
| Never | 511 (47.3) | 540 (50.0) |  |  | 133 (19.7) | 187 (27.1) |  |  | 378 (93.6) | 353 (90.5) |  |
| Missing | 0 (0.0) | 1 (0.1) |  |  | 0 (0.0) | 0 (0.0) |  |  | 0 (0.0) | 1 (0.3) |  |
| Alcohol consumption |  |  | <0.001 |  |  |  | <0.001 |  |  |  | <0.001 |
| Current | 641 (59.3) | 505 (46.7) |  |  | 478 (70.6) | 403 (58.3) |  |  | 163 (40.4) | 102 (26.2) |  |
| Former | 101 (9.3) | 163 (15.1) |  |  | 84 (12.4) | 123 (17.8) |  |  | 17 (4.2) | 40 (10.3) |  |
| Never | 339 (31.4) | 412 (38.1) |  |  | 115 (17.0) | 165 (23.9) |  |  | 224 (55.5) | 247 (63.3) |  |
| Missing | 0 (0.0) | 1 (0.1) |  |  | 0 (0.0) | 0 (0.0) |  |  | 0 (0.0) | 1 (0.3) |  |
| Regular exercise |  |  | <0.001 |  |  |  | <0.001 |  |  |  | <0.001 |
| Yes | 608 (56.2) | 387 (35.8) |  |  | 383 (56.6) | 254 (36.8) |  |  | 225 (55.7) | 133 (34.1) |  |
| No | 415 (38.4) | 694 (64.2) |  |  | 280 (41.4) | 437 (63.2) |  |  | 135 (33.4) | 257 (65.9) |  |
| Missing | 58 (5.4) | 0 (0.0) |  |  | 14 (2.1) | 0 (0.0) |  |  | 44 (10.9) | 0 (0.0) |  |
| Physical activity (MET-min/week) | 2524.4±2649.5 | 2124.3±2041.4 | <0.001 |  | 2823.2±2829.0 | 2290.4±2130.7 | <0.001 |  | 2025.1±2234.5 | 1830.9±1839.6 | 0.18 |
| Red meat intake (g/day) ^†^ | 58.09±42.50 | 48.08±35.74 | <0.001 |  | 59.91±45.04 | 51.37±38.18 | <0.001 |  | 55.05±37.71 | 42.25±30.14 | <0.001 |

^*^p-value was obtained by chi-squared and student’s t-test for continuous and categorical variables, respectively.

^†^Red meat intake was adjusted for total energy intake using residual method.

CRC, colorectal cancer; MET, metabolic equivalent of task.
