# Supplementary material for: Interactions between vitamin B2, the MTRR rs1801394 and MTR rs1805087 genetic polymorphisms, and colorectal cancer risk in a Korean population
Source: Epidemiol Health. 2024 Mar 11;46:e2024037. doi: 10.4178/epih.e2024037 (PMC11369566; doi:10.4178/epih.e2024037)
Supplement: Supplementary Material 4. — Association between the MTRR rs1801394 (A66G) and MTR rs1805087 (A2756G) genetic polymorphisms and CRC risk in the matched population [file epih-46-e2024037-Supplementary-4.docx]

Supplementary Material 4. Association between the *MTRR* rs1801394 (A66G) and *MTR* rs1805087 (A2756G) genetic polymorphisms and CRC risk in the matched population

|  | Colorectal cancer | | | | Proximal colon cancer | | | Distal colon cancer | | | Rectal cancer | | |
| --- | --- | --- | --- | --- | --- | --- | --- | --- | --- | --- | --- | --- | --- |
|  | No. of Controls (%) | No. of cases (%) | Model I, OR (95% CI) | Model II, OR (95% CI) | No. of cases (%) | Model I, OR (95% CI) | Model II, OR (95% CI) | No. of cases (%) | Model I, OR (95% CI) | Model II, OR (95% CI) | No. of cases (%) | Model I, OR (95% CI) | Model II, OR (95% CI) |
| rs1801394 |  |  |  |  |  |  |  |  |  |  |  |  |  |
| Co-dominant model |  |  |  |  |  |  |  |  |  |  |  |  |  |
| A/A | 576 (53.3) | 559 (51.7) | 1.00 | 1.00 | 190 (50.7) | 1.00 | 1.00 | 185 (52.3) | 1.00 | 1.00 | 180 (52.6) | 1.00 | 1.00 |
| G/A | 423 (39.1) | 435 (40.2) | 1.06 (0.89-1.27) | 1.50 (0.15-1.95) | 156 (41.6) | 1.12 (0.87-1.43) | 1.25 (0.95-1.63) | 140 (39.6) | 1.03 (0.80-1.33) | 1.15 (0.87-1.51) | 136 (39.8) | 1.03 (0.80-1.33) | 1.20 (0.90-1.59) |
| G/G | 82 (7.6) | 87 (8.1) | 1.09 (0.79-1.51) | 1.17 (0.73-1.86) | 29 (7.7) | 1.07 (0.68-1.69) | 1.04 (0.63-1.71) | 29 (8.2) | 1.10 (0.70-1.74) | 1.05 (0.64-1.72) | 26 (7.6) | 1.02 (0.63-1.63) | 0.98 (0.59-1.65) |
| Dominant model |  |  |  |  |  |  |  |  |  |  |  |  |  |
| A/A | 576 (53.3) | 559 (51.7) | 1.00 | 1.00 | 190 (50.7) | 1.00 | 1.00 | 185 (52.3) | 1.00 | 1.00 | 180 (52.6) | 1.00 | 1.00 |
| G/A + G/G | 505 (46.7) | 522 (48.3) | 1.06 (0.90-1.26) | 1.43 (1.12-1.83) | 185 (49.3) | 1.11 (0.88-1.41) | 1.21 (0.93-1.57) | 169 (47.7) | 1.04 (0.82-1.33) | 1.13 (0.87-1.47) | 162 (47.4) | 1.03 (0.81-1.31) | 1.16 (0.88-1.52) |
| Recessive model |  |  |  |  |  |  |  |  |  |  |  |  |  |
| A/A + G/A | 999 (92.4) | 994 (92.0) | 1.00 | 1.00 | 346 (92.3) | 1.00 | 1.00 | 325 (91.8) | 1.00 | 1.00 | 316 (92.4) | 1.00 | 1.00 |
| G/G | 82 (7.6) | 87 (8.1) | 1.07 (0.78-1.46) | 0.99 (0.63-1.56) | 29 (7.7) | 1.02 (0.66-1.59) | 0.95 (0.58-1.53) | 29 (8.2) | 1.09 (0.70-1.69) | 0.99 (0.62-1.60) | 26 (7.6) | 1.00 (0.63-1.59) | 0.91 (0.55-1.51) |
| rs1805087 |  |  |  |  |  |  |  |  |  |  |  |  |  |
| Co-dominant model |  |  |  |  |  |  |  |  |  |  |  |  |  |
| A/A | 814 (75.3) | 807 (74.7) | 1.00 | 1.00 | 283 (75.5) | 1.00 | 1.00 | 273 (77.1) | 1.00 | 1.00 | 245 (71.6) | 1.00 | 1.00 |
| G/A | 248 (22.9) | 253 (23.4) | 1.03 (0.84-1.26) | 0.77 (0.57-1.03) | 87 (23.2) | 1.01 (0.76-1.33) | 0.84 (0.62-1.14) | 75 (21.2) | 0.90 (0.67-1.21) | 0.75 (0.55-1.03) | 88 (25.7) | 1.18 (0.89-1.56) | 0.95 (0.69-1.29) |
| G/G | 19 (1.8) | 21 (1.9) | 1.12 (0.60-2.09) | 1.85 (0.72-4.72) | 5 (1.3) | 0.76 (0.28-2.05) | 0.96 (0.33-2.78) | 6 (1.7) | 0.94 (0.37-2.38) | 1.14 (0.42-3.10) | 9 (2.6) | 1.58 (0.70-3.53) | 1.80 (0.72-4.54) |
| Dominant model |  |  |  |  |  |  |  |  |  |  |  |  |  |
| A/A | 814 (75.3) | 807 (74.7) | 1.00 | 1.00 | 283 (75.5) | 1.00 | 1.00 | 273 (77.1) | 1.00 | 1.00 | 245 (71.6) | 1.00 | 1.00 |
| G/A + G/G | 267 (24.7) | 274 (25.4) | 1.04 (0.85-1.26) | 0.82 (0.62-1.09) | 92 (24.5) | 0.99 (0.75-1.30) | 0.85 (0.63-1.14) | 81 (22.9) | 0.91 (0.68-1.20) | 0.77 (0.57-1.05) | 97 (28.4) | 1.21 (0.92-1.59) | 0.99 (0.73-1.34) |
| Recessive model |  |  |  |  |  |  |  |  |  |  |  |  |  |
| A/A + G/A | 1062 (98.2) | 1060 (98.1) | 1.00 | 1.00 | 370 (98.7) | 1.00 | 1.00 | 348 (98.3) | 1.00 | 1.00 | 333 (97.4) | 1.00 | 1.00 |
| G/G | 19 (1.8) | 21 (1.9) | 1.11 (0.59-2.07) | 1.94 (0.76-4.93) | 5 (1.3) | 0.76 (0.28-2.04) | 1.00 (0.35-2.89) | 6 (1.7) | 0.96 (0.38-2.43) | 1.22 (0.45-3.31) | 9 (2.6) | 1.51 (0.68-3.37) | 1.83 (0.73-4.58) |
| Risk score of combination of two SNPs |  |  |  |  |  |  |  |  |  |  |  |  |  |
| 1-SD unit change |  |  | 1.04 (0.95-1.13) | 1.04 (0.94-1.15) |  | 1.03 (0.91-1.16) | 1.03 (0.91-1.18) |  | 1.00 (0.88-1.13) | 1.00 (0.87-1.14) |  | 1.07 (0.95-1.21) | 1.07 (0.93-1.23) |

Model I: crude model

Model II: adjusted for age, sex, body mass index, alcohol consumption, smoking status, marital status, occupation, education, family history of CRC, supplement use, monthly income, regular exercise, red meat intake, and total energy intake

CRC, colorectal cancer; OR, odds ratio; CI, confidence interval.
