# Supplementary material for: Interactions between vitamin B2, the MTRR rs1801394 and MTR rs1805087 genetic polymorphisms, and colorectal cancer risk in a Korean population
Source: Epidemiol Health. 2024 Mar 11;46:e2024037. doi: 10.4178/epih.e2024037 (PMC11369566; doi:10.4178/epih.e2024037)
Supplement: Supplementary Material 5. — . Interaction between vitamin B2 intake and the MTRR A66G and MTR A2756G genetic polymorphisms on CRC risk in the pre-matched population. [file epih-46-e2024037-Supplementary-5.docx]

Supplementary Material 5. Interaction between vitamin B_2_ intake and the *MTRR* A66G and *MTR* A2756G genetic polymorphisms on CRC risk in the pre-matched population.

| **Vitamin B_2_ (mg/d)** |  | | | |  | | | |  |
| --- | --- | --- | --- | --- | --- | --- | --- | --- | --- |
|  | **Q1** | **Q2** | **Q3** | **Q4** | **Q1** | **Q2** | **Q3** | **Q4** | **p for interaction^†^** |
| rs1801394 | **A/A** | | | | **G/A + G/G** | | | |  |
| All | Q1 (<0.95) | Q2 (0.95-1.18) | Q3 (1.18-1.43) | Q4 (≥1.43) | Q1 (<0.95) | Q2 (0.95-1.18) | Q3 (1.18-1.43) | Q4 (≥1.43) |  |
| No. of controls/cases | 259/181 | 266/175 | 268/119 | 274/84 | 248/159 | 240/162 | 237/109 | 233/92 |  |
| Model I, OR (95% CI) | 1.00 | 0.94 (0.72-1.23) | 0.64 (0.48-0.85) | 0.44 (0.32-0.60) | 0.92 (0.70-1.21) | 0.97 (0.73-1.27) | 0.66 (0.49-0.89) | 0.57 (0.42-0.77) | 0.28 |
| Model II, OR (95% CI) | 1.00 | 0.83 (0.60-1.14) | 0.74 (0.52-1.04) | 0.60 (0.41-0.88) | 0.90 (0.65-1.26) | 1.12 (0.80-1.57) | 0.84 (0.59-1.21) | 0.80 (0.55-1.17) | 0.22 |
| Male | Q1 (<0.90) | Q2 (0.90-1.12) | Q3 (1.12-1.35) | Q4 (≥1.35) | Q1 (<0.90) | Q2 (0.90-1.12) | Q3 (1.12-1.35) | Q4 (≥1.35) |  |
| No. of controls/cases | 156/104 | 156/121 | 171/82 | 179/54 | 152/97 | 152/103 | 137/68 | 129/62 |  |
| Model I, OR (95% CI) | 1.00 | 1.16 (0.83-1.64) | 0.72 (0.50-1.03) | 0.45 (0.31-0.67) | 0.96 (0.67-1.37) | 1.02 (0.72-1.45) | 0.75 (0.51-1.09) | 0.72 (0.49-1.07) | 0.07 |
| Model II, OR (95% CI) | 1.00 | 1.06 (0.69-1.61) | 0.69 (0.44-1.08) | 0.48 (0.30-0.78) | 0.85 (0.55-1.31) | 0.94 (0.61-1.46) | 0.88 (0.55-1.40) | 0.82 (0.50-1.34) | 0.02 |
| Female | Q1 (<1.03) | Q2 (1.03-1.30) | Q3 (1.30-1.60) | Q4 (≥1.60) | Q1 (<1.03) | Q2 (1.03-1.30) | Q3 (1.30-1.60) | Q4 (≥1.60) |  |
| No. of controls/cases | 100/73 | 108/59 | 100/43 | 97/23 | 98/65 | 90/65 | 99/36 | 101/26 |  |
| Model I, OR (95% CI) | 1.00 | 0.75 (0.48-1.16) | 0.59 (0.37-0.94) | 0.33 (0.19-0.56) | 0.91 (0.59-1.40) | 0.99 (0.64-1.54) | 0.50 (0.31-0.81) | 0.35 (0.21-0.60) | 0.94 |
| Model II, OR (95% CI) | 1.00 | 0.89 (0.52-1.53) | 1.06 (0.60-1.87) | 0.70 (0.36-1.35) | 1.26 (0.73-2.18) | 1.54 (0.89-2.65) | 0.99 (0.55-1.81) | 0.72 (0.38-1.34) | 0.72 |
| rs1805087 | **A/A** | | | | **G/A + G/G** | | | |  |
| All | Q1 (<0.95) | Q2 (0.95-1.18) | Q3 (1.18-1.43) | Q4 (≥1.43) | Q1 (<0.95) | Q2 (0.95-1.18) | Q3 (1.18-1.43) | Q4 (≥1.43) |  |
| No. of controls/cases | 371/252 | 375/258 | 380/162 | 398/135 | 136/88 | 131/79 | 125/66 | 109/41 |  |
| Model I, OR (95% CI) | 1.00 | 1.01 (0.81-1.27) | 0.63 (0.49-0.80) | 0.50 (0.39-0.64) | 0.95 (0.70-1.30) | 0.89 (0.64-1.23) | 0.78 (0.55-1.09) | 0.55 (0.37-0.82) | 0.28 |
| Model II, OR (95% CI) | 1.00 | 0.97 (0.74-1.28) | 0.69 (0.51-0.92) | 0.67 (0.49-0.92) | 0.74 (0.51-1.08) | 0.78 (0.53-1.15) | 1.00 (0.67-1.50) | 0.67 (0.42-1.07) | 0.16 |
| Male | Q1 (<0.90) | Q2 (0.90-1.12) | Q3 (1.12-1.35) | Q4 (≥1.35) | Q1 (<0.90) | Q2 (0.90-1.12) | Q3 (1.12-1.35) | Q4 (≥1.35) |  |
| No. of controls/cases | 220/152 | 230/168 | 236/106 | 249/89 | 88/49 | 78/56 | 72/44 | 59/27 |  |
| Model I, OR (95% CI) | 1.00 | 1.06 (0.79-1.41) | 0.65 (0.48-0.89) | 0.52 (0.38-0.71) | 0.81 (0.54-1.21) | 1.04 (0.70-1.55) | 0.89 (0.58-1.36) | 0.66 (0.40-1.09) | 0.07 |
| Model II, OR (95% CI) | 1.00 | 1.00 (0.71-1.43) | 0.67 (0.46-0.97) | 0.57 (0.38-0.84) | 0.66 (0.40-1.07) | 0.87 (0.53-1.41) | 0.99 (0.60-1.41) | 0.74 (0.40-1.37) | 0.02 |
| Female | Q1 (<1.03) | Q2 (1.03-1.30) | Q3 (1.30-1.60) | Q4 (≥1.60) | Q1 (<1.03) | Q2 (1.03-1.30) | Q3 (1.30-1.60) | Q4 (≥1.60) |  |
| No. of controls/cases | 147/102 | 149/94 | 141/61 | 152/35 | 51/36 | 49/30 | 58/18 | 46/14 |  |
| Model I, OR (95% CI) | 1.00 | 0.91 (0.63-1.31) | 0.62 (0.42-0.92) | 0.33 (0.21-0.52) | 1.02 (0.62-1.67) | 0.88 (0.53-1.48) | 0.45 (0.25-0.80) | 0.44 (0.23-0.84) | 0.94 |
| Model II, OR (95% CI) | 1.00 | 1.07 (0.68-1.67) | 1.01 (0.62-1.66) | 0.61 (0.36-1.05) | 0.97 (0.53-1.78) | 0.98 (0.51-1.85) | 0.71 (0.35-1.42) | 0.69 (0.31-1.51) | >0.999 |

Model I: crude model

Model II: adjusted for age, sex, body mass index, alcohol consumption, smoking status, marital status, occupation, education, family history of CRC, supplement use, monthly income, regular exercise, red meat intake, and total energy intake. The sex variable was excluded for the male and female groups.

**^†^**False discovery rate adjusted p-values.

CRC, colorectal cancer; OR, odds ratio; CI, confidence interval.
